# S-Adenosylmethionine Increases the Sensitivity of Human Colorectal Cancer Cells to 5-Fluorouracil by Inhibiting P-Glycoprotein Expression and NF-κB Activation

**DOI:** 10.3390/ijms22179286

**Published:** 2021-08-27

**Authors:** Laura Mosca, Martina Pagano, Luigi Borzacchiello, Luigi Mele, Annapina Russo, Giulia Russo, Giovanna Cacciapuoti, Marina Porcelli

**Affiliations:** 1 Department of Precision Medicine, University of Campania “Luigi Vanvitelli”, Via Luigi De Crecchio 7, 80138 Naples, Italy; laura.mosca@unicampania.it (L.M.); martina.pagano@unicampania.it (M.P.); luigi.borzacchiello@unicampania.it (L.B.); 2 Department of Pharmacy, University of Naples “Federico II”, Via Domenico Montesano 49, 80131 Naples, Italy; annapina.russo@unina.it (A.R.); giulia.russo@unina.it (G.R.); 3 Department of Experimental Medicine, University of Campania “Luigi Vanvitelli”, Via Luciano Armanni 5, 80138 Naples, Italy; luigi.mele@unicampania.it

**Keywords:** S-Adenosylmethionine, colorectal cancer, 5-Fluorouracil, multidrug resistance, P-glycoprotein, combination therapy

## Abstract

Colorectal cancer (CRC) is the second deadliest cancer worldwide despite significant advances in both diagnosis and therapy. The high incidence of CRC and its poor prognosis, partially attributed to multi-drug resistance and antiapoptotic activity of cancer cells, arouse strong interest in the identification and development of new treatments. S-Adenosylmethionine (AdoMet), a natural compound and a nutritional supplement, is well known for its antiproliferative and proapoptotic effects as well as for its potential in overcoming drug resistance in many kinds of human tumors. Here, we report that AdoMet enhanced the antitumor activity of 5-Fluorouracil (5-FU) in HCT 116^p53+/+^ and in LoVo CRC cells through the inhibition of autophagy, induced by 5-FU as a cell defense mechanism to escape the drug cytotoxicity. Multiple drug resistance is mainly due to the overexpression of drug efflux pumps, such as P-glycoprotein (P-gp). We demonstrate here that AdoMet was able to revert the 5-FU-induced upregulation of P-gp expression and to decrease levels of acetylated NF-κB, the activated form of NF-κB, the major antiapoptotic factor involved in P-gp-related chemoresistance. Overall, our data show that AdoMet, was able to overcome 5-FU chemoresistance in CRC cells by targeting multiple pathways such as autophagy, P-gp expression, and NF-κB signaling activation and provided important implications for the development of new adjuvant therapies to improve CRC treatment and patient outcomes.

## 1. Introduction

Colorectal cancer (CRC) is currently one of the most common malignancies worldwide, characterized by high mortality and morbidity in both men and women [1,2].

Survival rates and therapies currently used in the treatment of CRC may vary depending on several factors, including patient’s age, general health, tumor size and localization, and stage of tumor [1,2]. The ordinary approaches to treat CRC are chemotherapy, surgery, and radiotherapy [3,4,5]. The administration of systemic chemotherapies is the mainstay treatment to achieve an improvement in quality of life and survival in about 30% of cancer patients that present advanced and unresectable disease. The drug 5-fluorouracil (5-FU) has been the first chemotherapeutic choice for the treatment of CRC in the last few years [3,6,7]. The overall response rate to 5-FU in advanced CRC is limited to 10-15%. Although irinotecan and oxaliplatin are usually used in combination with 5-FU to improve survival, toxicity still increases. Indeed, despite initially impressive clinical responses, it is not uncommon to observe phenomena of intrinsic or acquired drug resistance and/or multidrug-resistance (MDR) events that lead to a loss of chemotherapy efficacy, CRC recurrence, and high mortality rate [3,4,6].

Over the years, one of the most important challenges has been to overcome resistance to first-line chemotherapy using various modulation strategies including association with new biological therapies such as anti-angiogenic, anti-EGFR, and multi-kinase inhibitors that have led to significant improvements in 5-FU-based combination regimens [3,4,6]. However, despite these advances in the treatment of CRC patients the prognosis is still poor, with median survivals that rarely reach 30 months. Therefore, new therapeutic strategies are immediately needed to combat drug resistance and increase drug response rates.

In this light, the methyl donor S-adenosyl-L-methionine (AdoMet), a multitargeted and safe FDA-approved natural compound, has been reported to work as a cancer-specific chemosensitizer in several types of human cancers [8,9,10,11,12,13,14] It has been reported that AdoMet was able to chemosensitize highly invasive MDA-MB-231 breast cancer cells to the action of 5-azacytidine, a DNA hypomethylating agent, resulting in the suppression of the prometastatic uPA gene expression and in the consequent inhibition of cell invasiveness [9]. In human cervical carcinoma, AdoMet in association with selenium compounds inhibited cell proliferation, migration, and adhesion via modulation of ERK and AKT signaling pathways [14]. In the hormone dependent CG5 breast cancer cell line, AdoMet synergized with doxorubicin in inhibiting proliferation via Fas/FasL signaling pathway activation [10,14]. In non-invasive MCF-7 breast cancer cells, the combined treatment of AdoMet with the autophagy inhibitor chloroquine, synergistically induced growth inhibition and apoptosis [8]. In pancreatic cancer cells AdoMet synergized with gemcitabine, an antineoplastic drug structurally similar to cytosine that inhibits DNA synthesis and ribonucleotide reductase activity, in inducing apoptosis and inhibiting invasion and migration through downregulation of JAK2/STAT3 signaling [11,14]. In head and neck squamous cancer cell lines, the combined treatment of AdoMet with cisplatin resulted in a strong synergism in inhibiting cell proliferation, enhancing apoptosis via intrinsic mechanism [13], and reducing cell migration [15]. Recently, in MDA-MB-231 cells and xenograft models of breast cancer, the combined therapeutic effects of AdoMet and decitabine, a hypomethylating agent, promoted a strong reduction in mammary tumor volume and lung metastasis [12,14].

Growing evidence accumulating in literature in recent years on the antiproliferative and proapoptotic activities exerted by AdoMet in colon cancer cells highlighted the pleiotropic effects of this eclectic multi-target sulfonium compound, evidencing its ability to modulate the genes responsible for cell invasion and metastasis [16,17,18,19,20,21,22]. In RKO and HT-29 colon cancer cells, AdoMet induced apoptosis by decreasing the expression of the gene encoding FLICE-like inhibitory protein, leading to caspase 8 activation and the consequent release of cytochrome c from the mitochondria [17]. AdoMet was able to inhibit tumor cell growth by reversing the hypomethylated status of c-Myc and H-ras promoters, thus suppressing the expression of these oncogenes [20]. In Balb/c mice after azoxymethane and dextran sulfate sodium treatment, AdoMet prevented the development of inflammation-induced colon cancer by azoxymethane and dextran sulfate sodium through inhibition of β-catenin, IL-6, pro-inflammatory, pro-growth, and proliferation signaling pathways [18]. In the highly invasive SW-620 colorectal cancer cell line, AdoMet reduced the metastatic process by hypermethylation of specific genes such as matrix metalloproteinase-2 and membrane type 1 matrix metalloproteinase [16]. While in SW480 and HCT 116 colon cancer cells with constitutively active β-catenin signaling, AdoMet inhibited β-catenin activity, preventing its access to the nuclear compartment. In RKO colon cancer cells expressing wild-type Wnt/β-catenin, AdoMet, improved GSK3β-mediated degradation of β-catenin by increasing the activity of protein phosphatase 2A [19]. Recently, in HT-29 and SW480 CRC cells, AdoMet inhibited the progression of cancer cells by modulating gene expression. Indeed, AdoMet reduced cell number and increased senescence by inducing cell cycle arrest at S-phase and by downregulating multiple epithelial-mesenchymal transition-related genes in both cell lines. In particular, in SW480 cells, AdoMet promoted the activation of DNA repair processes through γ-H2AX elevation [21]. More recently, our research group demonstrated that AdoMet may overcome uL3-mediated drug resistance in p53-deleted HCT116 colon cancer cells by inducing cell cycle arrest at the S-phase, inhibition of autophagy, increase of reactive oxygen species generation, and finally activation of the apoptotic pathway [22]. Altogether, these findings clearly highlight the potential of the methyl donor AdoMet to reduce CRC progression. In spite of this, the ability of the sulfonium compound to restore the cytotoxic activity of classic anticancer drugs and to reverse the MDR process in CRC has not been thoroughly investigated. It is known that resistance to chemotherapy has been correlated with overexpression of P-glycoprotein (P-gp), a member of the ATP-binding cassette (ABC) superfamily of membrane transporters whose expression is closely associated with the nuclear factor kappa-light-chain-enhancer of activated B cells (NF-kB) signaling pathway [23,24,25]. 

In this study, we evaluated for the first time the ability of AdoMet to enhance the antitumor activity of 5-FU in HCT 116 ^p53+/+^ and in LoVo colon cancer cells by overcoming MDR and we investigated AdoMet-induced modulation of p-glycoprotein expression and NF-kB signaling downregulation. Our findings may have important implications for the development of new adjuvant therapies based on this natural and safe compound to improve CRC treatment and patient outcome in the future. 

## 2. Results

### 2.1. AdoMet Inhibited HCT 116^p53+/+^ and LoVo Colorectal Cells Viability

To study the antiproliferative activity of AdoMet we first evaluated its effect on the cell viability of HCT 116^p53+/+^ and LoVo cells, both expressing p53. Notably, in a previous paper [22] we demonstrated that AdoMet reduced the proliferation of HCT 116^p53−/−^ and uL3∆HCT 116^p53−/−^ cells although, according to the literature, the lack of p53 expression is usually associated with loss of p53-dependent tumor-suppressive functions, making tumor cells more resistant to chemotherapy treatments [26].

**Figure 1:**
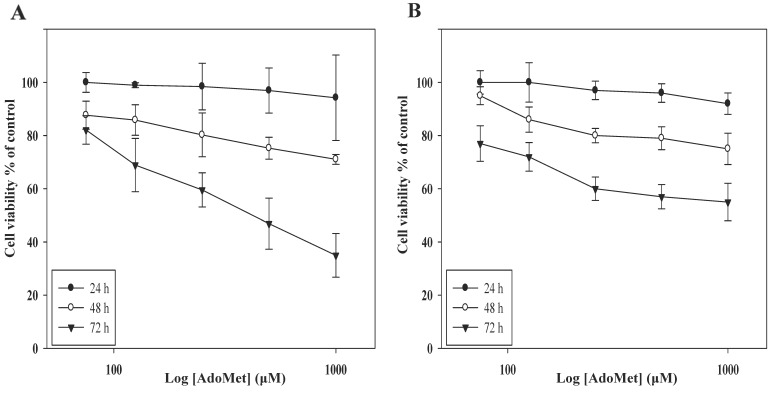
Effect of AdoMet on colorectal cancer cell viability. HCT 116^p53+/+^ (**A**) and LoVo (**B**) cells were treated or not (control) with increasing amounts of AdoMet (72–1000 µM) for 24, 48 and 72 h, then cell viability was assessed by MTT assay. Data represent the average of three independent experiments; error bars depict the standard deviation (SDs).

As reported in Figure 1, statistically significant time- and dose-dependent growth inhibition occurred when cells were incubated with increasing AdoMet concentrations from 72 to 1000 μM for different times. The effect was evaluated by MTT assay and resulted in IC50 values of 500 μM at 72 h in both cell lines, in good agreement with the data reported in p53-deleted cells [22]. These results demonstrate that the antiproliferative effect of AdoMet in CRC cells is independent of p53 status and that AdoMet is able to inhibit the CRC proliferation with an efficacy comparable to that described in other types of human cancer cells [8,10].

### 2.2. AdoMet Potentiated 5-FU-Induced Apoptosis in HCT 116^p53+/+^ and LoVo Colorectal Cell Lines

**Figure 2:**
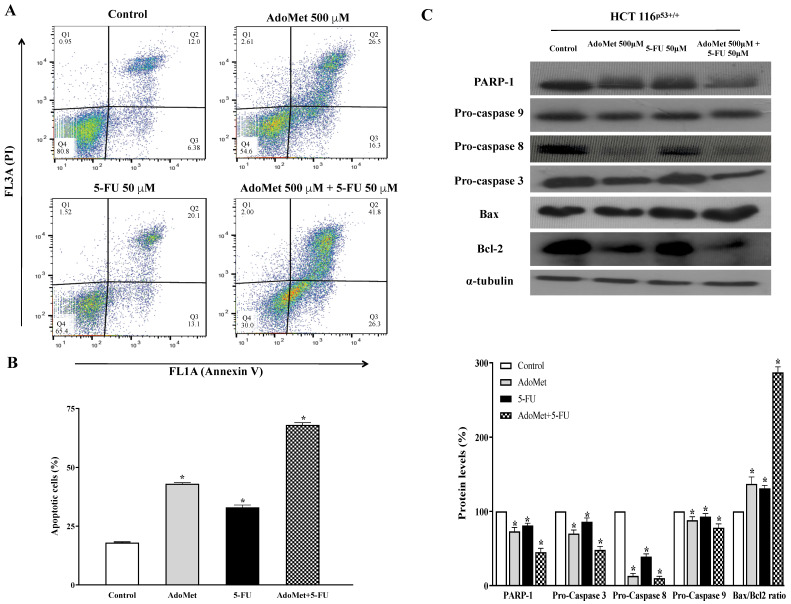
Effect of AdoMet, 5-FU and their combination on apoptosis in HCT 116^p53+/+^ cells. (**A**) Cells were treated with 5-FU 50 µM for 24 h with and without 48 h pretreatment with 500 µM AdoMet. Apoptosis was then evaluated by FACS analysis. Representative dot plots of both Annexin V-FITC and PI-stained cells. The different quadrants show the percentage of cells: viable cells, lower left (Q4); early apoptotic cells, bottom right (Q3); late apoptotic cells, top right (Q2); nonviable necrotic cells, upper left (Q1). For each sample, 2 × 10^4^ events were acquired. Analysis was carried out by triplicate determination of at least 3 separate experiments. (**B**) The histogram plot shows, for each single treatment, the percentage of apoptotic cells in the Q2 and Q3 quadrants. Data represent the average of three independent experiments. (**C**) The protein levels of PARP-1, pro-caspase 9, pro-caspase 8, pro-caspase 3, Bax, and Bcl-2 were detected by Western blot analysis using the total cell lysates. The house-keeping protein α-tubulin was used as loading control. The graph reports the relative densitometric analyses expressed as percentage of untreated control (100%). Error bars represent the standard deviation. * *p* < 0.05 versus untreated cells. The images are representative of three immunoblotting analyses obtained from three independent experiments. Full-length blots are shown in Figure S1.

**Figure 3:**
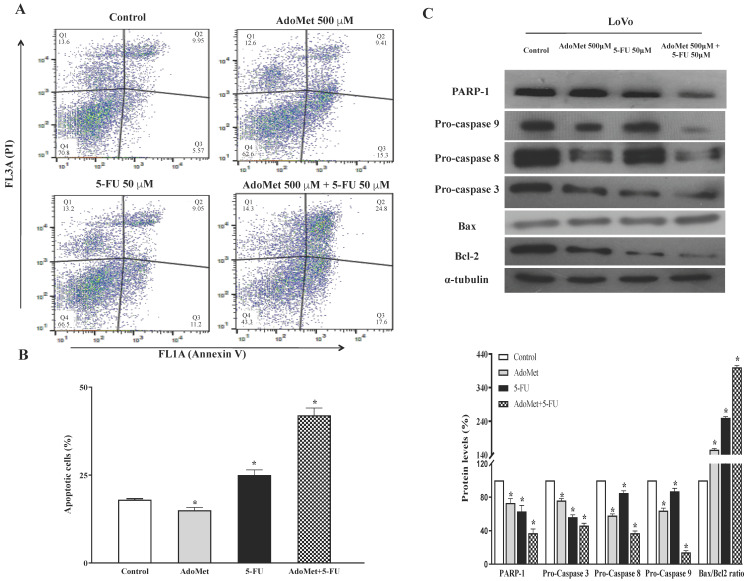
Effect of AdoMet, 5-FU and their combination on apoptosis in LoVo cells. (**A**) Cells were untreated with 5-FU 50 µM for 24 h with and without 48 h pretreatment with 500 µM AdoMet. Apoptosis was then evaluated by FACS analysis. Representative dot plots of both Annexin V-FITC and PI-stained cells. The different quadrants report the percentage of cells: viable cells, lower left (Q4); early apoptotic cells, bottom right (Q3); late apoptotic cells, top right (Q2); nonviable necrotic cells, upper left (Q1). For each sample, 2 × 10^4^ events were acquired. Analysis was carried out by triplicate determination of at least 3 separate experiments. (**B**) The histogram plot shows, for each single treatment, the percentage of apoptotic cells in the Q2 and Q3 quadrants. Data represent the average of three independent experiments. (**C**) The protein levels of PARP-1, pro-caspase 9, pro-caspase 8, pro-caspase 3, Bax, and Bcl-2 were detected by Western blot analysis using the total cell lysates. The house-keeping protein α-tubulin was used as loading control. The graph reports the relative densitometric analyses expressed as percentage of untreated control (100%). Error bars represent the standard deviation. * *p* < 0.05 versus untreated cells. The images are representative of three immunoblotting analyses obtained from three independent experiments. Full-length blots are shown in Figure S1.

In order to investigate whether the growth inhibition upon AdoMet treatment was associated with the induction of apoptotic cell death, CRC cells were treated with AdoMet 500 μM and the apoptotic process was evaluated after 72 h by FACS analysis. As shown in Figure 2A and Figure 3A, AdoMet induced a significant increase of apoptotic cells compared to the control with percentage values of 24% and 15% in HCT 116^p53+/+^ and LoVo cells, respectively, indicating that apoptosis represented the underlying mechanism of AdoMet-mediated growth inhibition in CRC cells. 

Treatment with 5-FU is known to improve survival in various types of cancers and is one of the most widely used drugs for the treatment of advanced CRC, with the greatest impact reported in the palliative and adjuvant settings [3,6,7]. Significant harmful side-effects are known to be produced by 5-FU and often the onset of drug resistance in cancer cells leads to the failure of chemotherapy.

To assess whether AdoMet may enhance the sensitivity of CRC cells to 5-FU we evaluated the effects of an AdoMet/5-FU combination on apoptotic cell death by FACS analysis. HCT 116^p53+/+^ and LoVo cells were pre-treated with AdoMet 500 µM for 48 h before treatment with 5-FU 50 µM for a further 24 h and the apoptotic process was evaluated after double labeling with Annexin V and PI. Flow cytometric analysis revealed that the combined treatment with AdoMet and 5-FU improved the proapoptotic effect of 5-FU in both cell lines. The effect was more pronounced in HCT 116^p53+/+^ cells where the apoptotic rate significantly increased from 15% to about 50% (Figure 2A,B) while in LoVo cells the percentage of apoptotic cells increased from 9% to 27% evidencing a greater drug resistance of this cell line (Figure 3A,B). These data suggest that CRC cells were more sensitive to treatment with 5-FU in combination with AdoMet than those treated with 5-FU alone, confirming that AdoMet is able to potentiate the cytotoxic effects of 5-FU.

In order to identify the mechanisms of apoptosis-mediated cell death, we monitored by Western blot analysis the expression level of the key markers of apoptotic cascade, such as procaspases 3, 8, 9, and PARP-1 involved in DNA damage repair, as well as Bax and Bcl-2, two mitochondria-associated modulators of apoptosis involved in the release of cytochrome C at the beginning of apoptosis. The balance of these pro- and antiapoptotic members of Bcl-2 gene family has been envisaged as the determinant of the functional integrity of the mitochondrial outer membrane and is thought one of the main mechanisms regulating intrinsic apoptotic process in mammalian cells [27]. Figure 2C and Figure 3C show that AdoMet, 5-FU, and their combination induced a decrease of pro-caspases and PARP-1 levels, more evident in combination treatments than after administration of the single drug, indicating that the apoptotic process occurs via a caspase-dependent mechanism. The increase of the Bax/Bcl-2 ratio observed in treated cells compared with the control indicated that the apoptotic process in HCT 116^p53+/+^ and LoVo cells might occur through the mitochondrial pathway. Figure 2C and Figure 3C also show that AdoMet caused significant activation of procaspase 8 in both cell lines, suggesting that the extrinsic apoptotic pathway could also play a role in AdoMet-induced inhibition of CRC cell growth. 

Altogether, the data indicated that AdoMet was able to sensitize HCT 116^p53+/+^ and LoVo cells to the antiproliferative effects of 5-FU by enhancing intrinsic and extrinsic apoptotic cell death.

### 2.3. AdoMet Inhibited 5-FU-Induced Autophagy in HCT 116^p53+/+^ and LoVo Colorectal Cancer Cells

In order to evaluate whether activation of apoptosis parallels another cell death mechanism, we assessed the onset of autophagy in CRC.

It has been widely reported in literature that CRC chemoresistance is related to increased autophagic flux [28]. Autophagy, an important homeostatic cellular recycling mechanism, is now emerging as a crucial player in the development of MDR by acting as a defense mechanism for cancer cells to escape from the cytotoxicity of chemotherapeutics. Thus, inhibition of autophagy can resensitize resistant tumor cells and enhance the effect of chemotherapy drugs [28].

**Figure 4:**
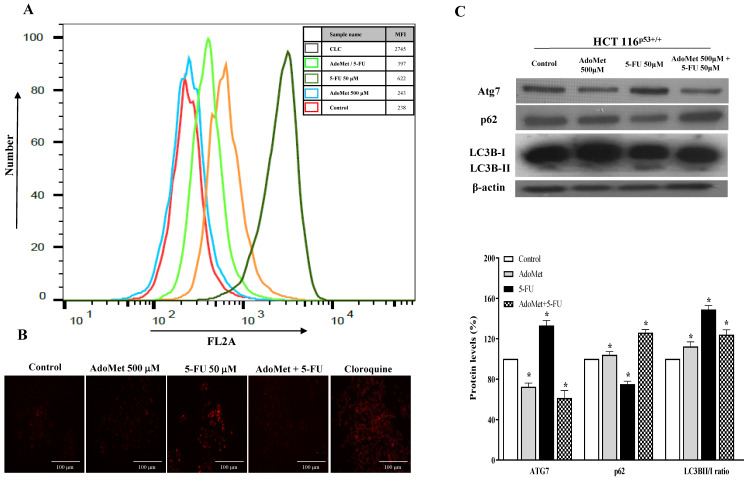
Effects of AdoMet, 5-FU, and their combination on the autophagic process in HCT 116^p53+/+^ cells. Cells were treated with 5-FU 50 µM for 24 h with and without pretreatment with 500 µM AdoMet for 24 h. Cells were then incubated with LTR and analyzed by flow cytometry (**A**) and fluorescence microscopy (**B**). Chloroquine was used as a positive control. For the quantitative evaluation of autophagy, the FlowJo software was used to calculate median fluorescence intensities (MFI) by the formula (MFI-treated/MFI-control). At least 2 × 10^4^ events were acquired in log mode. Analysis was carried out by triplicate determination on at least three separate experiments. (**C**) The protein contents of Atg7, p62, and LC3B-II/I were detected by Western blot analysis using the total cell lysates. The house-keeping protein β-actin was used as loading control. The graph shows the relative densitometric analyses, expressed as percentage of untreated control (100%). Error bars represent the standard deviation, * *p* < 0.05 versus untreated cells. The images are representative of three immunoblotting analyses obtained from three independent experiments. Full-length blots are shown in Figure S2.

**Figure 5:**
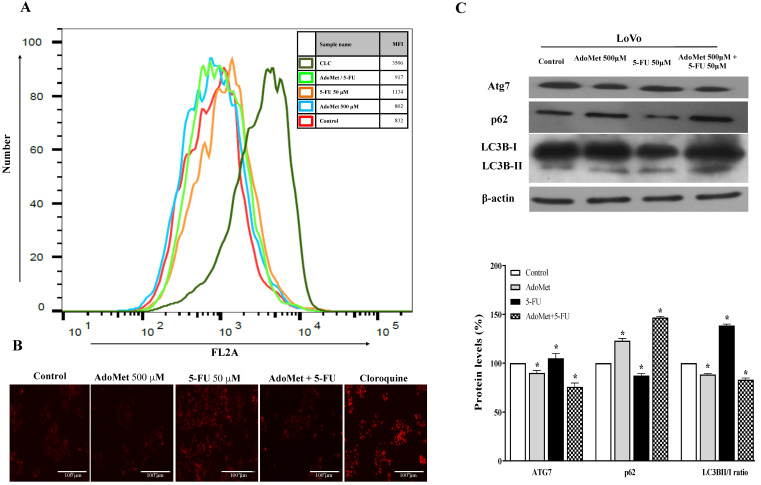
Effects of AdoMet, 5-FU, and their combination on the autophagic process in LoVo cells. Cells were treated with 5-FU 50 µM for 24 h with and without pretreatment with 500 µM AdoMet for 24 h. Cells were then incubated with LTR and analyzed by flow cytometry (**A**) and fluorescence microscopy (**B**). Chloroquine was used as a positive control. For the quantitative evaluation of autophagy, the FlowJo software was used to calculate median fluorescence intensities (MFI) by the formula (MFI-treated/MFI-control). At least 2 × 10^4^ events were acquired in log mode. Analysis was carried out by triplicate determination on at least three separate experiments. (**C**) The protein contents of Atg7, p62, and LC3B-II/I were detected by Western blot analysis using the total cell lysates. The β-actin was used as loading control. The graph shows the relative densitometric analyses expressed as percentage of untreated control (100%). Error bars represent the standard deviation, * *p* < 0.05 versus untreated cells. The images are representative of three immunoblotting analyses obtained from three independent experiments. Full-length blots are shown in Figure S2.

To examine the autophagic responses of HCT 116^p53+/+^ and LoVo cells to 5-FU treatment and to explore the chemosensitizing role of AdoMet to 5-FU cytotoxicity and the underlying mechanisms, the cells were treated with 50 µM 5-FU for 24 h with or without 24 h pretreatment with 500 mM AdoMet, and the autophagic process was then analyzed by FACS and fluorescence microscopy after staining with the vital dye LTR, a fluorescent probe for labeling and monitoring acidic organelles in living cells [8]. Quantitative analysis by flow cytometry evidenced a significant increase of the autophagic flux in both HCT 116^p53+/+^ (Figure 4A) and LoVo (Figure 5A) cells treated with 5-FU compared to untreated or AdoMet-pretreated cells that was accompanied by an increased formation of red dotted acidic vacuoles (Figure 4B and Figure 5B). These data were confirmed by Western blot analysis of some markers widely used to monitor autophagy such as p62, Atg7, and LC3BI/II (Figure 4C and Figure 5C). The p62 protein targets ubiquitinated proteins and delivers them to autophagosomes for degradation. Since p62 is degraded during the autophagic process, its level can be utilized as a good indicator of the autophagic flux. Indeed, the amount of intracellular p62 decreases when autophagy is induced. On the contrary, a corresponding accumulation of the protein could be observed when autophagy is inhibited [29,30,31]. Atg7 is an essential protein for cell degradation. During the initiation of autophagy Atg7 functions as an E1-like ligase for ubiquitin-like proteins such as Atg8 and Atg12, which are needed for the autophagic vacuole formation [29,30,31]. Finally, LC3B is the main marker of autophagy, and its amount correlates with the extent of autophagosome formation. LC3B is normally placed in the cytosol (LC3B-I) but it becomes lipidated, cleaved (LC3B-II), and incorporated into autophagosomal membranes after autophagic induction [29,30,31]. We found that 5-FU downregulated p62, enhanced Atg7 and caused LC3I to LC3II conversion in both HCT 116^p53+/+^ (Figure 4C) and LoVo (Figure 5C) cells, thus inducing the activation of autophagy as a potential mechanism to promote chemoresistance. Conversely, pretreatment of cells with AdoMet inhibited 5-FU-induced activation of the autophagic process as detected by red staining (Figure 4A,B and Figure 5A,B), and reverted the changes brought by 5-FU on the expressions of p62 and Atg7 and on the LC3BI I/I ratio (Figure 4C and Figure 5C). The obtained results indicate that the inhibition of 5-FU-induced autophagy could represent one of the mechanisms utilized by AdoMet to chemosensitize HCT 116^p53+/+^ and LoVo CRC cells to 5-FU toxicity and to overcome autophagy-related chemoresistance. 

### 2.4. AdoMet Inhibited P-gp Expression and NF-κB Acetylation Induced by 5-FU in HCT 116^p53+/+^ and LoVo Colorectal Cells

Several tumor cells overexpress P-gp causing failure of chemotherapy due to an increased efflux of drug molecules from the cancer cells and thereby resulting in a decrease in intracellular drug concentration. This mechanism ultimately determines drug resistance [32,33].

**Figure 6:**
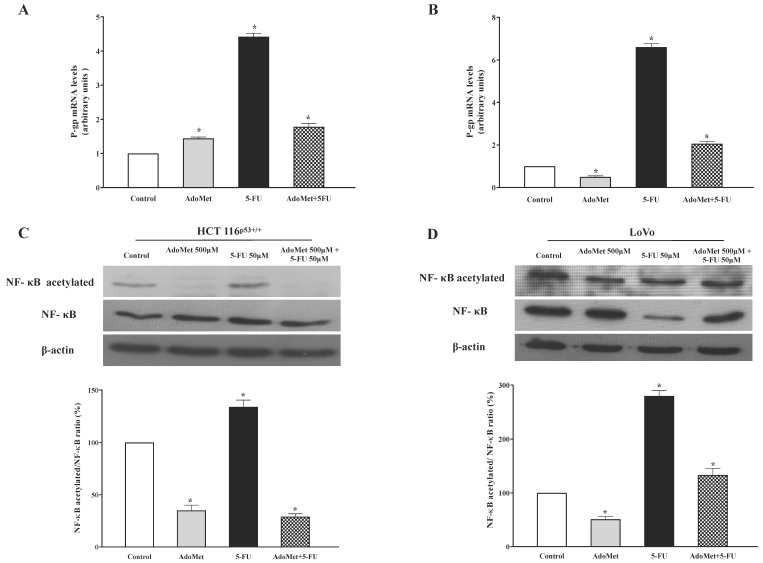
Effect of AdoMet and/or 5-FU on the levels of P-gp, NF-κB, and its acetylated form in HCT 116^p53+/+^ and LoVo cells. Cells were treated with 5-FU 50 µM for 24 h, with and without pretreatment with 500 µM AdoMet for 48 h. Total RNA was extracted and cDNA was synthesized by qRT-PCR. The graphs show the mRNA levels of P-gp in HCT 116^p53+/+^ (**A**) and LoVo (**B**) cells normalized to GAPDH mRNA. Data represent the average of three independent experiments. Western blot assay was used for the expression of NF-κB and acetylated NF-κB in HCT 116^p53+/+^ (**C**) and LoVo (**D**) cells, respectively. β-actin was used as loading control. Graphs show the relative densitometric analyses, expressed as percentage of untreated control (100%). Error bars represent the standard deviation, * *p* < 0.05 versus untreated cells. The images are representative of three immunoblotting analyses obtained from at least three independent experiments. Full-length blots are shown in Figure S3.

To explore the molecular mechanisms underlying the anticancer effect of AdoMet in CRC cells and to evaluate if the modulation of P-gp is involved in AdoMet-induced resistance to 5-FU, we performed a qRT-PCR analysis of P-gp after 24 h incubation of HCT 116^p53+/+^ and LoVo cells with 50 µM 5-FU, with and without 48 h pretreatment with 500 µM AdoMet. The results obtained show no changes in the transcriptional expression levels of P-gp after treatment with AdoMet alone, while a strong upregulation of P-gp of about 3.5-fold and 5.6-fold higher than the control was detected in HCT 116^p53+/+^ and LoVo cells, respectively, after treatment with 5-FU (Figure 6A,B) Interestingly, in the combined treatment AdoMet strongly reduced the upregulation of P-gp induced by 5-FU, bringing the P-gp mRNA levels back to values approximately 0.9- and 1.0-fold higher than the control in HCT 116^p53+/+^ and LoVo cells respectively. The findings indicate that AdoMet could revert 5-FU-induced upregulation of P-gp and highlighted P-gp as a target of the chemosensitizing effect of AdoMet in CRC cells.

The inhibition of P-gp expression is often related to the NF-kB degradation [23,24,25]. NF-κB is one of the main antiapoptotic factors involved in chemoresistance. Accordingly, elevated NF-κB activity has been detected in drug-resistant tumor cells [23,34]. 

To explore whether the ability of AdoMet to revert 5-FU-induced upregulation of P-gp is associated with the modulation of NF-κB signaling, the expression levels of NF-κB and its acetylated form were analyzed by Western blotting. The results shown in Figure 6C,D highlight high levels of acetylated NF-κB as well as an increased NF-κB acetylated/NF-κB protein ratio in cells treated with 5-FU, while a strong reduction of these values was observed in cells treated with AdoMet or with AdoMet/5-FU combination. These findings suggest that the suppression of NF-κB activation could represent one of the mechanisms by which AdoMet downregulated the expression of P-gp. 

Overall, the data indicate that the inhibitory effect of AdoMet on P-gp expression and its ability to revert the 5-FU-induced chemoresistance of HCT 116^p53+/+^ and LoVo CRC cells were closely associated with the inhibition of NF-κB signaling activation. The results obtained also suggested that a combined therapy with AdoMet and 5-FU may be an efficient way to achieve antitumor synergy in treating and preventing drug resistance in CRC cells.

## 3. Discussion

The antimetabolite drug 5-FU exerts its anticancer effects by inhibiting thymidylate synthase activity, preventing methylation of deoxyuridine monophosphate and impairing DNA synthesis and repair processes [3,6,7]. Currently, 5-FU is the most important chemotherapeutic molecule used for the treatment of CRC. Despite advances in systemic therapy, the development of drug resistance, toxicity, and its associated adverse effects limit the clinical application of 5-FU and are the main cause of CRC progression. Various mechanisms can explain 5-FU resistance including evasion of apoptosis, induction of autophagy, overexpression of drug transporters, and activation of epithelial-to-mesenchymal transition. Epigenetic changes mediated by 5-FU and microRNA dysregulation also play important roles in CRC carcinogenesis and are strongly involved in the acquisition of 5-FU resistance [3,4,7]. 

The potential of AdoMet for cancer prevention has been widely investigated [13,14,22] and much evidence in recent years has highlighted this naturally-occurring multifunctional sulfonium compound as a promising chemosensitizing agent in various cancer types [8,9,10,11,12,13,14]. Interestingly, AdoMet is one of the most widely studied epigenetic regulators and a well-documented modulator of noncoding RNAs involved in oncogenic functions [35,36,37,38]. The development of combination therapy with natural compounds able to synergize with chemotherapy drugs without causing significant toxic side-effects may represent a promising approach to increase 5-FU sensitivity and prevent drug resistance in CRC cells. In line with this suggestion, the new findings of the present study show that pretreatment of HCT 116^p53+/+^ and LoVo colorectal cancer cells with AdoMet overcame 5-FU resistance and provided experimental evidence on the underlying mechanisms.

Further deepening our studies on the antitumor effect of AdoMet in CRC [22] show that the sulfonium compound effectively caused a time- and dose-dependent inhibition of HCT 116^p53+/+^ and LoVo cell viability; allowing us to take AdoMet into consideration as a new natural agent for CRC targeting.

Autophagy and apoptosis play crucial roles in mediating cell survival and cell death and much evidence highlights a crosstalk relationship between these two cellular processes in modulating the development and progression of CRC [31,39]. Cancer cells frequently counteract these processes to achieve drug-resistance. Induction of apoptosis is known as a therapeutic target for CRC and its failure represents an important factor in the poor response of CRC cells to chemotherapy. Of particular note, the efficacy of drugs targeting apoptosis-related signaling such as death receptor 5 (DR5), Bcl-2 family proteins, and caspase activity have been tested in pre-clinical and clinical trials in the treatment of CRC [40]. In line with this evidence, we showed that AdoMet, as a single treatment, induced cell death in HCT 116^p53+/+^ and LoVo cells by triggering both extrinsic and intrinsic apoptosis as suggested by activation of caspase 3, 8, and 9, and by PARP cleavage. AdoMet also modulated the Bax/Bcl-2 ratio by downregulating the antiapoptotic Bcl-2 and upregulating proapoptotic Bax expression. Notably, the Bcl-2 family proteins, key regulators of intrinsic apoptotic pathways, have been implicated in CRC initiation and progression and in chemoresistance. Recently, the potential of BH3 mimetics, small molecular antagonists of antiapoptotic Bcl-2 family members, has emerged as a therapeutic opportunity to target intrinsic apoptotic pathways in CRC [27]. We found that AdoMet potentiated the cytotoxic effect of 5-FU in both CRC cell lines by synergistically enhancing the proapoptotic activity of the drug suggesting that combined AdoMet/5-FU treatment may represent a strategy to overcome drug resistance and reduce side effects in CRC.

In tumor cells, autophagy acts a “double-edged sword”. On the one hand, in specific conditions autophagy displays a cytoprotective function in cancer therapy and is associated with drug resistance mechanisms that represent a clinical obstacle to the success of cancer treatments [29,31,41]. Interestingly, autophagy inhibitors, such as chloroquine and hydroxychloroquine, have already been clinically approved, and have been utilized in combined chemotherapeutic treatments to target autophagic pathways in CRC [42]. On the other hand, autophagy can function as a tumor suppressor mechanism similar to apoptosis depending on tissue type and its specific microenvironment, stage of tumor development, and degree of autophagy activity [29,30,31]. Growing evidence indicates that inhibition of autophagy potentiates the cytotoxic effects of 5-FU in CRC and that the anticancer activity of 5-FU is enhanced in a synergistic manner by autophagy inhibitors [43]. Interestingly, it has been reported that the combined treatment of 5-FU with phytochemicals synergistically induced apoptosis in CRC cells and that the observed synergistic effect could be related to inhibition of the autophagic process [44]. Notably, we provided evidence that single-treatment with 5-FU activated cytoprotective autophagy in human colon carcinoma HCT 116^p53+/+^ and LoVo cells inducing drug resistance. Conversely, AdoMet pretreatment was able to revert 5-FU-induced autophagy as indicated by the reduced LC3BII/I ratio and by the decreased Atg7 and the increased p62 levels, while concomitantly enhancing apoptotic cell death. Notably, AdoMet-induced activation of caspase 8 observed in 5-FU-treated HCT 116^p53+/+^ and LoVo cells is in agreement with literature data indicating that p62 accumulation, caused by autophagy impairment, promoted caspase 8 activation during cisplatin-induced apoptosis in ovarian cancer cells resulting in enhanced sensitivity to the chemotherapeutic drug [45].

On this basis, it is conceivable that combining an antitumor agent with cytoprotective autophagy effects, such as 5-FU, with AdoMet, a multifaceted compound able to act simultaneously as an autophagy inhibitor and apoptosis inducer could be a good opportunity to overcome drug resistance and to enhance 5-FU antitumor therapy in CRC cells.

The P-gp transporter, also referred to as ATP-binding cassette subfamily B member 1 (ABCB1) or multidrug resistance protein 1 (MDR1), is among the most clinically important ABC transporters [32,33]. Physiologically, P-gp is constitutively expressed in cells with specific barrier functions, such as the intestine where it is involved in the detoxification of xenobiotics, while in cancer cells P-gp functions as an energy-driven efflux pump which induces a significant reduction in intracellular accumulation of drugs, decreasing the efficacy of chemotherapy and ultimately causing MDR. P-gp has been found to be overexpressed in a variety of cancers, including the colon, where it has been shown to correlate with an overall poor chemotherapy response and prognosis [23,24,25,26,27,28,29,30,31,32,33]. Until now, P-gp inhibitors previously identified as potential co-therapeutics for treatment of MDR have not been successful in clinical trials [25,26,27,28,29,30,31,32,33,34,35,36,37,38,39,40,41,42,43,44,45,46]. More recently, growing interest has been paid to developing natural products-based agents to revers P-gp-mediated multidrug resistance in cancer cells because of their low toxicity, selective behavior, and ability to target various signal transduction pathways [32,47]. P-gp-mediated MDR can be reversed by inhibiting the pump function of the transporter or by preventing its expression. Since inhibiting the transporter function could cause harmful effects leading to accumulation of chemotherapeutic drugs in non-target normal tissues, the downregulation of P-gp gene expression in tumor tissues is considered a better approach for the reversal of MDR. In addition, 5-FU therapy failure is associated with the increased expression of P-gp levels in CRC cells. Consistent with this view, we demonstrated that AdoMet restored 5-FU-induced upregulation of P-gp in HCT 116^p53+/+^ and LoVo cells, highlighting the potential of this multifunctional natural compound to overcome P-gp-mediated chemoresistance in CRC cells.

Overexpression of the ABCB1 gene in response to many transcription factors, including NF-κB, has been demonstrated in MDR cell lines [23,24]. Direct binding of NF-κB to the proximal promoter region of ABCB1 gene activates its transcription in MDR cells thus modulating the molecular mechanisms underlying P gp mediated MDR [24].

NF-κB is a ubiquitous transcription factor that upon induction regulates the expression of different target genes to modulate the immune system, inflammation, angiogenesis, survival, and proliferation of cells [23,34]. In mammals, NF-κB comprises five subunits that can bind to promoter regions of target genes as homodimers or heterodimers. The most common dimer consists of two different subunits, p65(RelA) and p50 which are required for activation and nuclear translocation. The nuclear function of NF-κB is regulated in part through reversible acetylation of its p65(RelA) subunit [48]. NF-κB is one of the major antiapoptotic and chemoresistance-related factors. Its constitutive or aberrant activation is a feature of a variety of solid tumors, including colorectal cancer where the NF-κB-signaling pathway functions as a key regulator of cell proliferation, apoptosis, angiogenesis, inflammation, metastasis, and drug resistance, suggesting that NF-κB inhibitors could be helpful in cancer therapies [49,50]. Acetylation of RelA on K310 is required for the full transcriptional potential of NF-κB, regulating its stability and preventing its methylation to lysine 314/315 which is important for the ubiquitination and degradation of Nf-κB. The abolition of the acetylation of lysine 310 decreases the transcriptional activity of NF-κB and, in turn, P-gp activation [48]. Since the expression of the multidrug transporter P-gp was found to be NF-kB-dependent it is conceivable that the co-administration of chemotherapeutic agents and NF-κB inhibitors could decrease P-gp expression and restore chemosensitivity in CRC cells. In line with this hypothesis we showed that the expression levels of acetylated NF-κB were significantly suppressed by AdoMet in HCT 116^p53+/+^ and LoVo cells and that pretreatment with AdoMet resulted in a notable decrease of acetylated NF-κB/NF-κB ratio in both 5-FU-treated cell lines, indicating that AdoMet could downregulate the expression of P-gp and reverse the MDR in CRC cells, partly at least, by suppressing the activation of the NF-κB signaling pathway. 

The reported findings are in line with previously published works on the effects of natural compounds in CRC therapy either in vitro, on colorectal cell lines, or in vivo studies on experimental models [51], and support the view that combination chemotherapy based on the administration of natural compounds, in association with conventional chemotherapeutics, could represent one of the most effective strategies for the treatment of numerous types of cancers.

Natural compounds, due to their ability to target multiple signaling pathways have been found able to increase the efficacy of standard chemotherapy by overcoming drug resistance and by reducing toxicity and unwanted side-effects. Interestingly, the selective toxic effects exerted by several natural compounds and their synergistic behavior in combined therapy allow us to diminish the burden on the patient’s organism by replacing part of the dose of a conventional chemotherapeutic with a natural substance with effects targeted mainly on cancer cells. According to the reported ability of AdoMet to reinforce the effective drug concentration, intensify the combined effect of both administered therapeutics, or exert cytotoxic effects specifically on tumor cells, the sulfonium compound could be considered one of the most promising natural molecules for improving combination therapy and minimizing the adverse effects associated with conventional chemotherapy.

Advanced drug delivery systems, as nanotechnologies, are emerging in order to deliver larger amounts of a therapeutic agent to the tumor, to increase the solubility and/or bioavailability of natural and synthetic compounds while limiting their systemic exposure and reducing systemic toxicity. Few studies are available in the literature about the formulation of AdoMet-loaded nanoparticles and their use as an environmentally sensitive vehicle suitable for controlling AdoMet delivery [52,53]. Based on the experimental evidence, the design of innovative technologies utilizing nanovectors incorporating AdoMet, alone or in combination with chemotherapeutic drugs, could open new avenues for improving bioavailability, reducing toxicity, and achieving selective cancer cell targeting and could be taken into consideration for future clinical applications in CRC management.

In conclusion, the ability of AdoMet in potentiating the cytotoxic effect of 5-FU by sensitizing CRC cells to be more responsive to the chemotherapeutic drug and its capacity to overcome the multidrug chemoresistance of CRC cells by targeting P-gp transporter add a further piece of information to the mosaic of antitumor activities of this natural compound, one that has emerged in the last decades as a fascinating molecule able to modulate several cancer-related molecular mechanisms, suggesting its possible application as an adjuvant in cancer management.

## 4. Materials and Methods

### 4.1. Materials

The drug 5-FU was purchased from Sigma-Aldrich (St. Louis, MO, USA). Bovine serum albumin (BSA), fetal bovine serum (FBS), Dulbecco’s modified Eagle’s medium (DMEM), F-12K Nutrient Mix (1X), phosphate-buffered saline (PBS), and trypsin-EDTA were bought from Gibco (Grand Island, NY, USA). Chloroquine (CLC), radioimmunoprecipitation assay buffer (RIPA buffer), propidium iodide (PI), and 3-(4,5-dimethylthiazol-2-yl)-2,5-diphenyltetrazolium bromide (MTT) were purchased from Sigma-Aldrich (St. Louis, MO, USA). Tissue culture dishes were purchased from Corning (Corning, NY, USA). AdoMet was obtained from New England Biolabs, prepared in a solution of 5 mM H2SO4 and 10% ethanol, filtered, and stored at 4 °C until use. LysoTracker-Red (LTR) and the Annexin V-fluorescein isothiocyanate (V-FITC) Apoptosis detection kits were provided by eBioscience (San Diego, CA, USA). Monoclonal antibodies to pro-caspase 9, pro-caspase 8, poly- (ADP ribose) polymerase 1 (PARP-1), Bcl-2, Bax, Atg7, β-actin, α-tubulin, and polyclonal antibodies to pro-caspase 3, LC3BI/II, p62, and NF-κB were purchased from Cell Signaling Technology (Danvers, MA, USA). Polyclonal antibody to NF-κB (acetyl K310) was purchased from Abcam (Cambridge, UK). Horse radish peroxidase (HRP)-conjugated goat anti-mouse (GxMu-003-DHRPX), and HRP-conjugated goat anti-rabbit (GtxRb-003- DHRPX) secondary antibodies were obtained from ImmunoReagents Inc. (Raleigh, NC, USA). High capacity cDNA reverse transcription kit was purchased from Applied Biosystems (Foster City, CA, USA). TRIzol RNA isolation reagents, SYBR™ Green PCR Master Mix and qRT-PCR primers: P-gp and glyceraldehyde 3-phosphate dehydrogenase (GAPDH) were obtained from Thermo Fisher Scientific (Waltham, Massachusetts, USA). All buffers and solutions were prepared with milliQ water. All reagents were at the purest commercial grade.

### 4.2. Cell Cultures and Drug Treatments

HCT 116^p53+/+^ and LoVo cells were provided by the American Type Culture Collection (ATCC Manassas, VA, USA). Cells were cultured at 37 °C in a 5% CO_2_ humidified atmosphere and grown in DMEM and F-12K Nutrient Mix (1X) respectively, both supplemented with 10% FBS, 2 mM L-glutamine, and penicillin-streptomycin 50 U/mL. Subconfluent cells were seeded in tissue culture plates and treated with AdoMet 500 µM and/or 5-FU 50 µM for 24, 48, and 72 h.

### 4.3. Cell Viability Assays

HCT 116^p53+/+^ and LoVo cells were plated in serum-enriched media in 96-well plates at the proper density. After 24 h incubation, the cells were treated with increasing concentrations of AdoMet (from 72 to 1000 µM) for 24, 48, and 72 h. Cell viability was assessed by adding MTT as previously reported [22]. The absorbance values of the solution in each well were measured at 570 nm using a Bio-Rad IMark microplate reader (Bio-Rad Laboratories, Milan, Italy). All experiments were carried out in quadruplicate. Cell viability was expressed as the percentage of absorbance values of treated samples with respect to the absorbance of the control (100%).

### 4.4. Flow Cytometry Analysis of Apoptosis

HCT 116^p53+/+^ and LoVo cells were seeded in 6-well plates, starved overnight, pretreated with 500 µM AdoMet for 72 h and treated with 5-FU 50 µM for 24 h, alone or in combination. Cells were harvested by trypsinization and washed twice with PBS. Annexin V-FITC was used in combination with the vital PI dye to differentiate the apoptotic cells (Annexin V-positive, PI-positive) from the necrotic ones (Annexin V-negative, PI-positive) as previously reported [54]. Briefly, the cells were resuspended in 200 µL of Binding Buffer 1X and incubated with 5 µL of Annexin V and 10 µL of PI (20 µg/mL) for 30 min at room temperature, as reported on the datasheet. The detection of viable cells, early apoptotic, late apoptotic, and necrotic cells was performed by BD FACS Aria III (Becton& Dickinson, Mountain View, CA, USA). For each sample, 20,000 events were recorded. Analysis was carried out by triplicate determination on at least three separate experiments.

### 4.5. Autophagy Detection by LysoTracker-Red Staining

HCT 116^p53+/+^ and LoVo cells were seeded in 6-well plates, pretreated with 500 µM AdoMet for 48 h and then treated with 5-FU 50 µM for 24 h, using CLC as positive control. LTR was added to each well for 20 min at 37 °C at the final concentration of 0.1 µM in medium. Subsequently, cells were washed with PBS and observed by fluorescence microscopy where snapshot images were captured to examine the autophagic process. Cell fluorescence intensity was then analyzed by flow cytometry. Briefly, the cells were detached by incubation with EDTA-trypsin, washed twice with PBS, and collected by centrifugation. For the quantitative assessment of LTR, FlowJo software was used to calculate the median fluorescence intensities (MFI) by the formula (MFI-treated/MFI-control), where MFI-treated is the fluorescence intensity of cells treated with the various compounds and MFI-control is the fluorescence intensity of the untreated cells. For each sample, 20,000 events were acquired. Analysis was carried out by triplicate determination in at least three separate experiments.

### 4.6. Protein Extraction and Western Blot Analysis

HCT 116^p53+/+^ and LoVo cells were harvested by trypsinization and lysed using 100 µL of RIPA buffer, as previously described [55]. After incubation on ice for 30 min, the samples were centrifuged at 18,000× *g* in an Eppendorf microcentrifuge for 30 min at 4 °C, and the supernatant was recovered. The protein concentration was determined and compared to standard BSA curve. Equal amounts of cell proteins were separated by sodium dodecyl sulfate-polyacrylamide gel electrophoresis (SDS-PAGE) and electrotransferred to nitrocellulose membranes by Trans blot turbo (BIO-RAD). The membranes were washed in 10 mM Tris-HCl, pH 8.0, 150 mM NaCl, 0.05% Tween 20 (TBST), and blocked with TBST supplemented with 5% nonfat dry milk. The membranes were then incubated with different primary antibodies in TBST and 5% nonfat dry milk, followed by incubation with HRP-conjugated secondary antibodies. All primary antibodies were used at a dilution of 1:1000 and 1:500; all secondary antibodies were used at a dilution of 1:5000 and 1:2000. Blots were developed using enhanced chemiluminescence detection reagents ECL (Cyanagen, Bologna, Italy) and exposed to X-ray film. All films were scanned by using ImageJ software (National Institutes of Health, Bethesda, MD, USA).

### 4.7. RNA Isolation, Reverse Transcription, and qRT-PCR

Total RNA was isolated from cultured HCT 116^p53+/+^ and LoVo cells treated with AdoMet 500 µM and/or 5-FU 50 µM, by using the TRIzol RNA isolation reagents, according to manufacturer’s instructions. Afterward, single-stranded cDNA was synthesized from total RNA samples by using the high-capacity cDNA reverse transcription kit. The expression of P-gp transcript was determined independently by quantitative real-time PCR (qRT-PCR), using SYBR Green PCR Master Mix (Applied Biosystems, Foster City, CA, USA). To normalize total RNA samples, GAPDH was selected as an appropriate constitutively expressed endogenous control. Relative expression of the transcripts was measured by using ViiA7™ Real-Time PCR software (Applied Biosystems, Foster City, CA, USA). The qRT-PCR primer sequences are as follows: P-gp, (Forward 5′-GCTGTCAAGGAAGCCAATGCCT-3′ and Reverse 5′-TGCAATGGCGATCCTCTGCTTC-3′; GAPDH, (Forward 5′-GGAGTCAACGGATTTGGTCG-3′ and Reverse 5′-CTTCCCGTTCTCAGCCTTGA-3′).

### 4.8. Statistical Analysis

Experiments were performed at least three times with replicate samples. Data are expressed as mean ± standard deviation (SD). *p*-values were determined using unpaired two-tailed Student’s *t*-test. A value of * *p* < 0.05 was considered statistically significant.

## Supplementary Materials

The following are available online at https://www.mdpi.com/article/10.3390/ijms22179286/s1.
