# Supplementary material for: S-Adenosylmethionine Increases the Sensitivity of Human Colorectal Cancer Cells to 5-Fluorouracil by Inhibiting P-Glycoprotein Expression and NF-κB Activation
Source: Int J Mol Sci. 2021 Aug 27;22(17):9286. doi: 10.3390/ijms22179286 (PMC8431578; doi:10.3390/ijms22179286)
Supplement: Supplementary file 1 [file ijms-22-09286-s001.zip › ijms-1350836 supplementary.pdf]

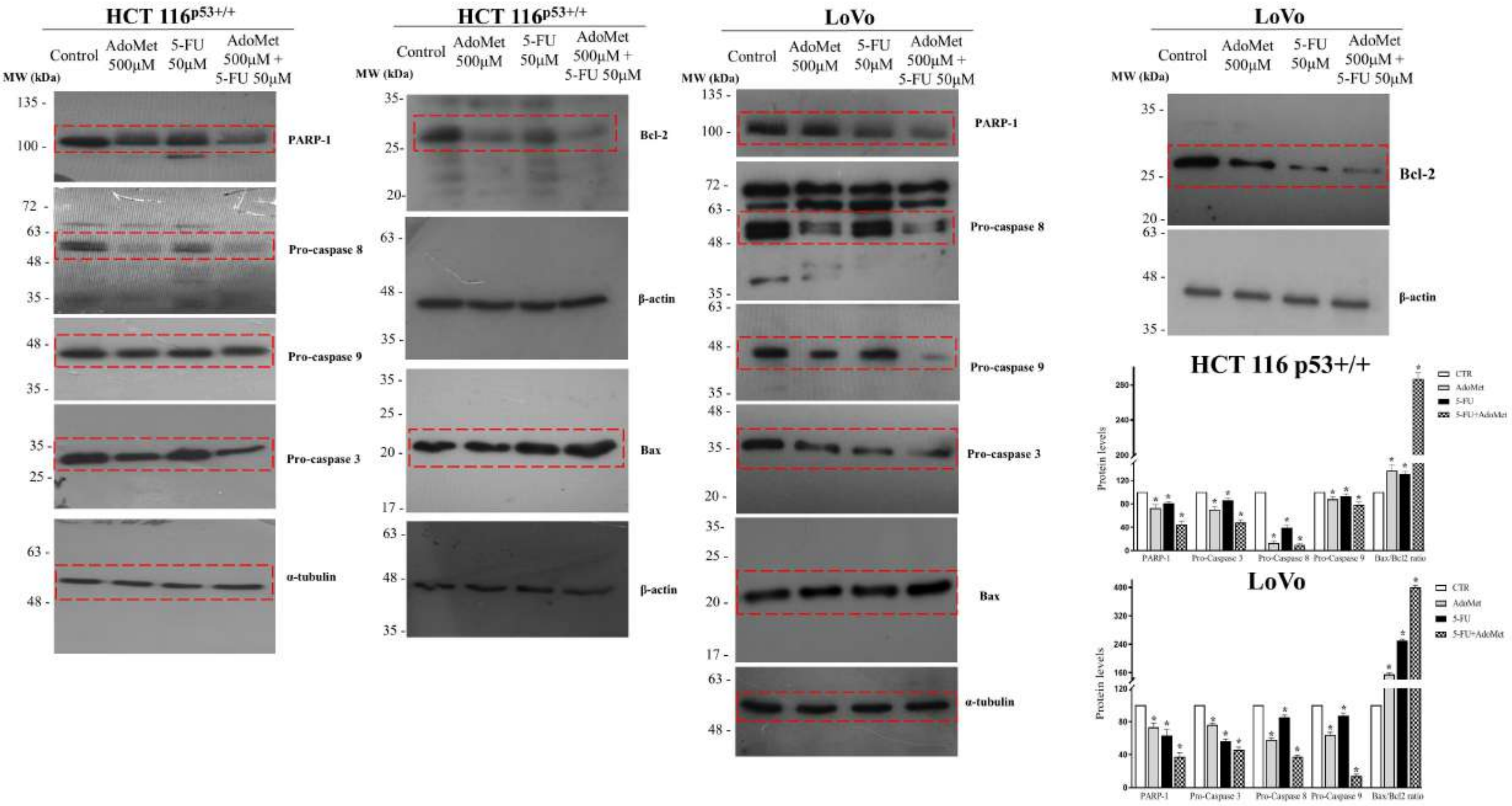

Figure S1. Effect of AdoMet, 5-FU, and combined treatments on apoptosis in HCT 116$^{p53+/+}$ and LoVo colon cancer cells. The cropped blots are used in the main figures (Figure 2 and 3).

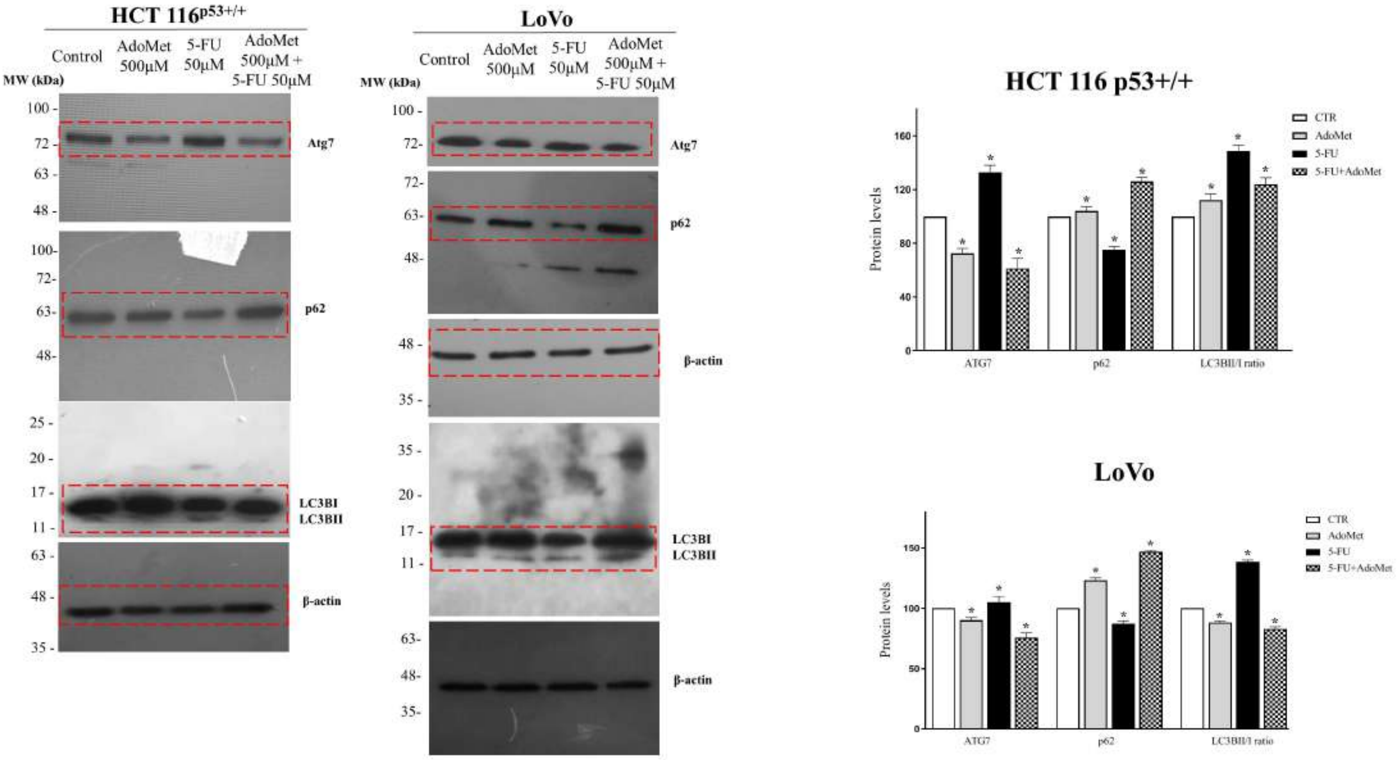

Figure S2. Effect of AdoMet 500 µM, 5-FU 50 µM, and combined treatments on the autophagy-related markers in HCT 116$^{p53+/+}$ and LoVo colon cancer cells. The cropped blots are used in the main figures (Figure 4 and 5).

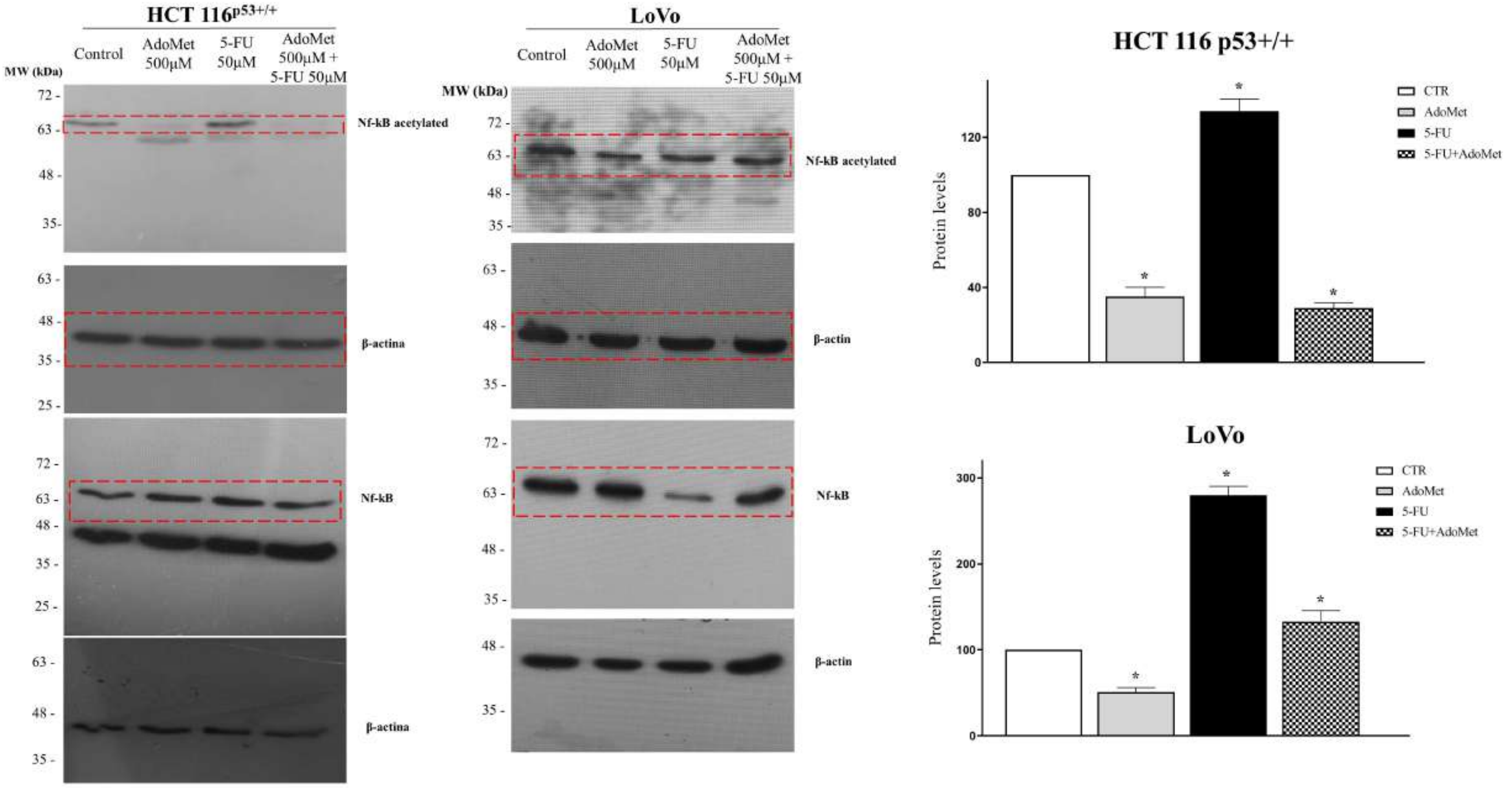

Figure S3. Effect of AdoMet 500 μM, 5-FU 50 μM, and combined treatments on Nf-kB and acetylated Nf-kB in HCT 116$^{p53+/+}$ and LoVo colon cancer cells. The cropped blots are used in the main figures (Figure 6).
