# Supplementary material for: S-Adenosylmethionine Increases the Sensitivity of Human Colorectal Cancer Cells to 5-Fluorouracil by Inhibiting P-Glycoprotein Expression and NF-κB Activation
Source: Int J Mol Sci. 2021 Aug 27;22(17):9286. doi: 10.3390/ijms22179286 (PMC8431578; doi:10.3390/ijms22179286)
Supplement: Supplementary file 1 [file ijms-22-09286-s001.zip › ijms-1350836 supplementary.pptx]

## Slide 1
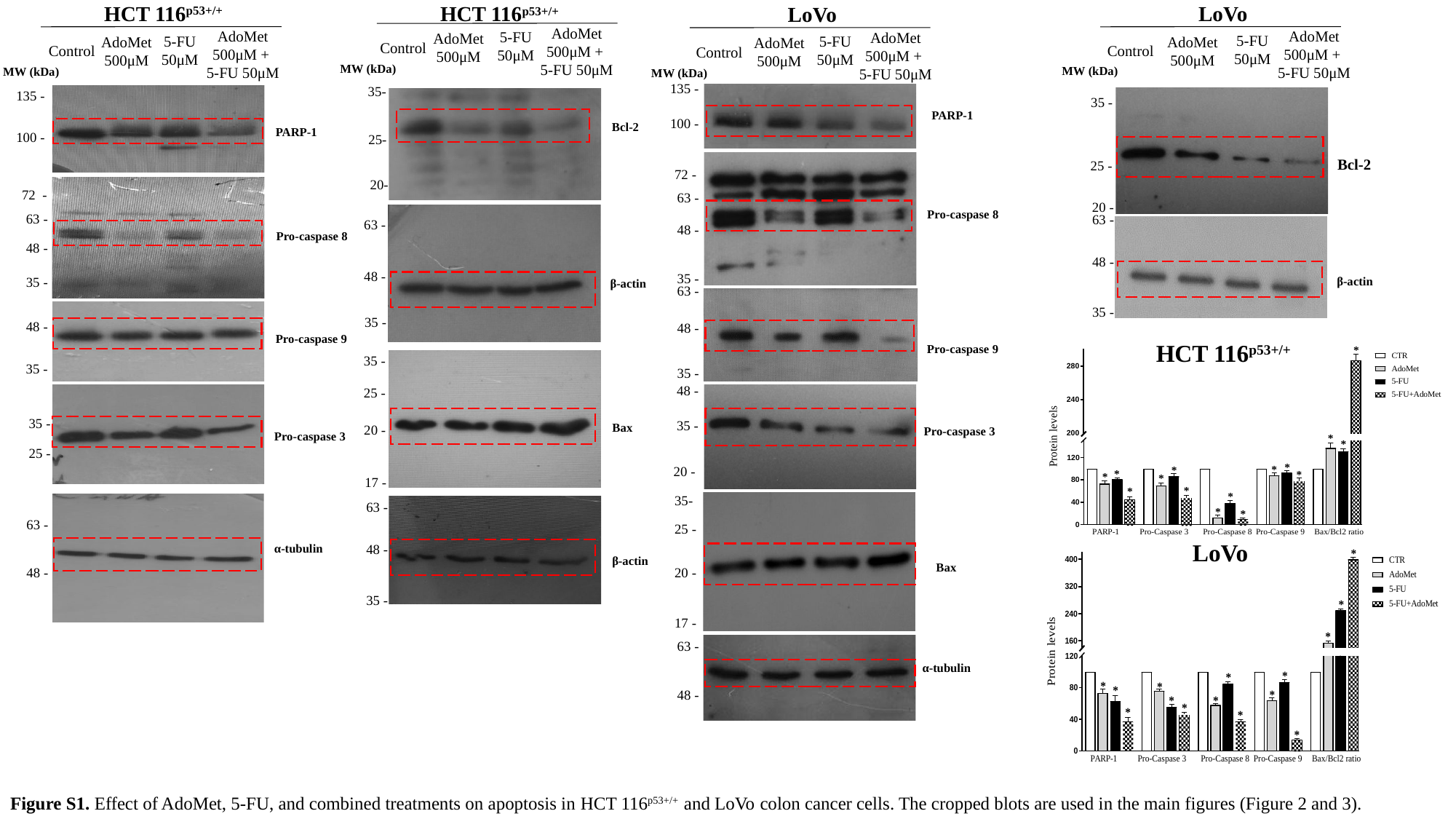

LoVo
HCT 116p53+/+
HCT 116p53+/+
LoVo
AdoMet 500μM +
5-FU 50μM
AdoMet 500μM +
5-FU 50μM
AdoMet 500μM +
5-FU 50μM
5-FU 50μM
AdoMet 500μM +
5-FU 50μM
AdoMet 500μM
5-FU 50μM
5-FU 50μM
5-FU 50μM
AdoMet 500μM
AdoMet 500μM
AdoMet 500μM
Control
Control
Control
Control
MW (kDa)
MW (kDa)
MW (kDa)
MW (kDa)
135 -
 35-
135 -
 35 -
 PARP-1
100 -
Bcl-2
PARP-1
100 -
 25-
Bcl-2
 25 -
 72 -
 20-
 72 -
63 -
 20 -
Pro-caspase 8
63 -
63 -
63 -
48 -
Pro-caspase 8
48 -
48 -
48 -
35 -
35 -
β-actin
β-actin
63 -
35 -
35 -
48 -
48 -
HCT 116 p53+/+
Pro-caspase 9
HCT 116p53+/+
Pro-caspase 9
 35 -
35 -
35 -
 48 -
 25 -
35 -
 35 -
Bax
 20 -
Pro-caspase 3
Pro-caspase 3
25 -
 20 -
 17 -
 35-
63 -
63 -
 25 -
LoVo
48 -
α-tubulin
β-actin
Bax
48 -
 20 -
35 -
 17 -
63 -
α-tubulin
48 -
Figure S1. Effect of AdoMet, 5-FU, and combined treatments on apoptosis in HCT 116p53+/+ and LoVo colon cancer cells. The cropped blots are used in the main figures (Figure 2 and 3).

## Slide 2
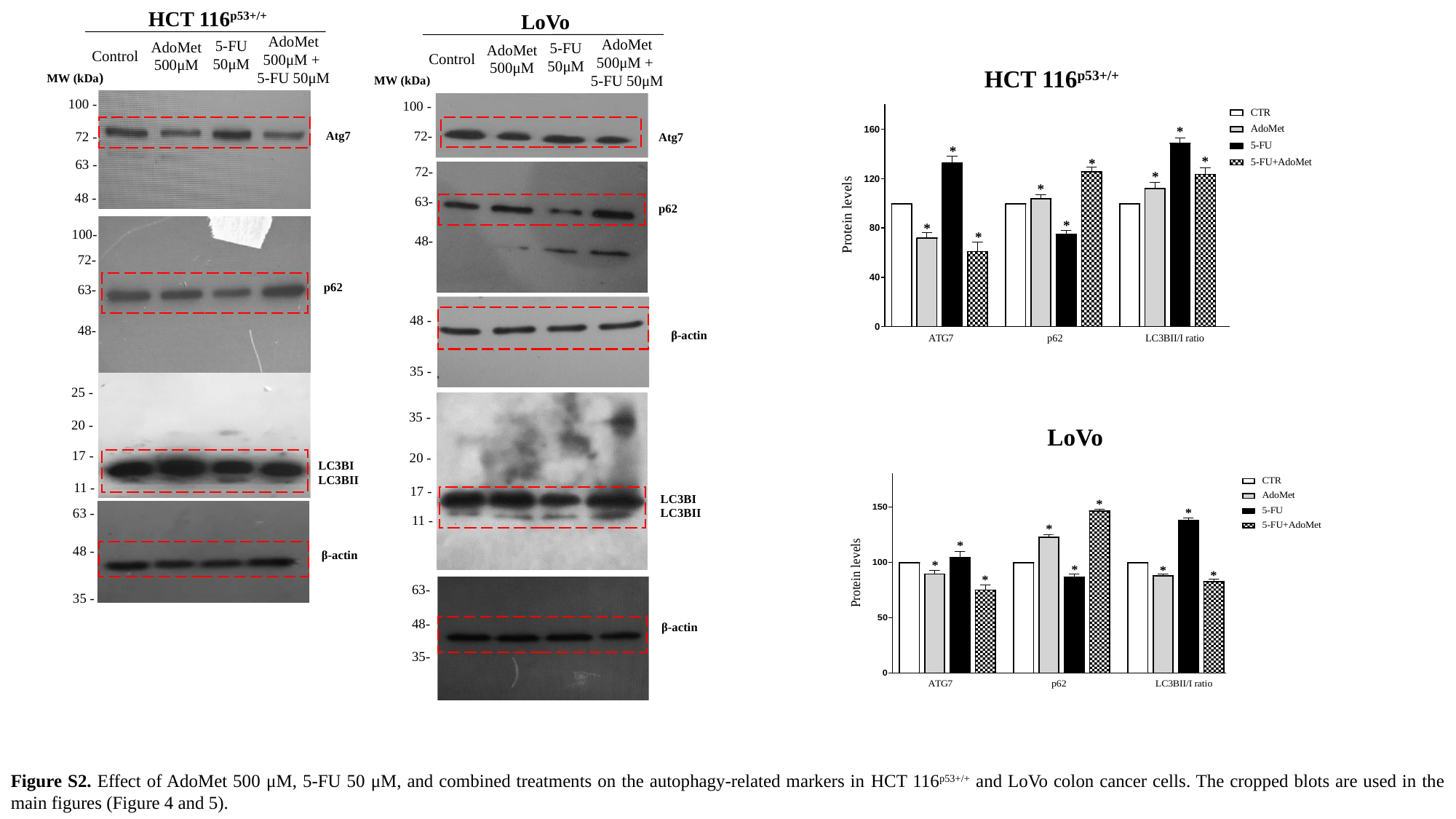

HCT 116p53+/+
LoVo
AdoMet 500μM +
5-FU 50μM
AdoMet 500μM +
5-FU 50μM
5-FU 50μM
AdoMet 500μM
5-FU 50μM
AdoMet 500μM
Control
Control
HCT 116p53+/+
MW (kDa)
MW (kDa)
100 -
100 -
 72-
Atg7
72 -
Atg7
63 -
 72-
48 -
 63-
p62
 100-
 48-
 72-
p62
 63-
48 -
 48-
β-actin
35 -
25 -
35 -
20 -
LoVo
17 -
20 -
LC3BI
LC3BII
11 -
17 -
LC3BI
LC3BII
63 -
11 -
48 -
β-actin
 63-
35 -
 48-
β-actin
 35-
Figure S2. Effect of AdoMet 500 μM, 5-FU 50 μM, and combined treatments on the autophagy-related markers in HCT 116p53+/+ and LoVo colon cancer cells. The cropped blots are used in the main figures (Figure 4 and 5).

## Slide 3
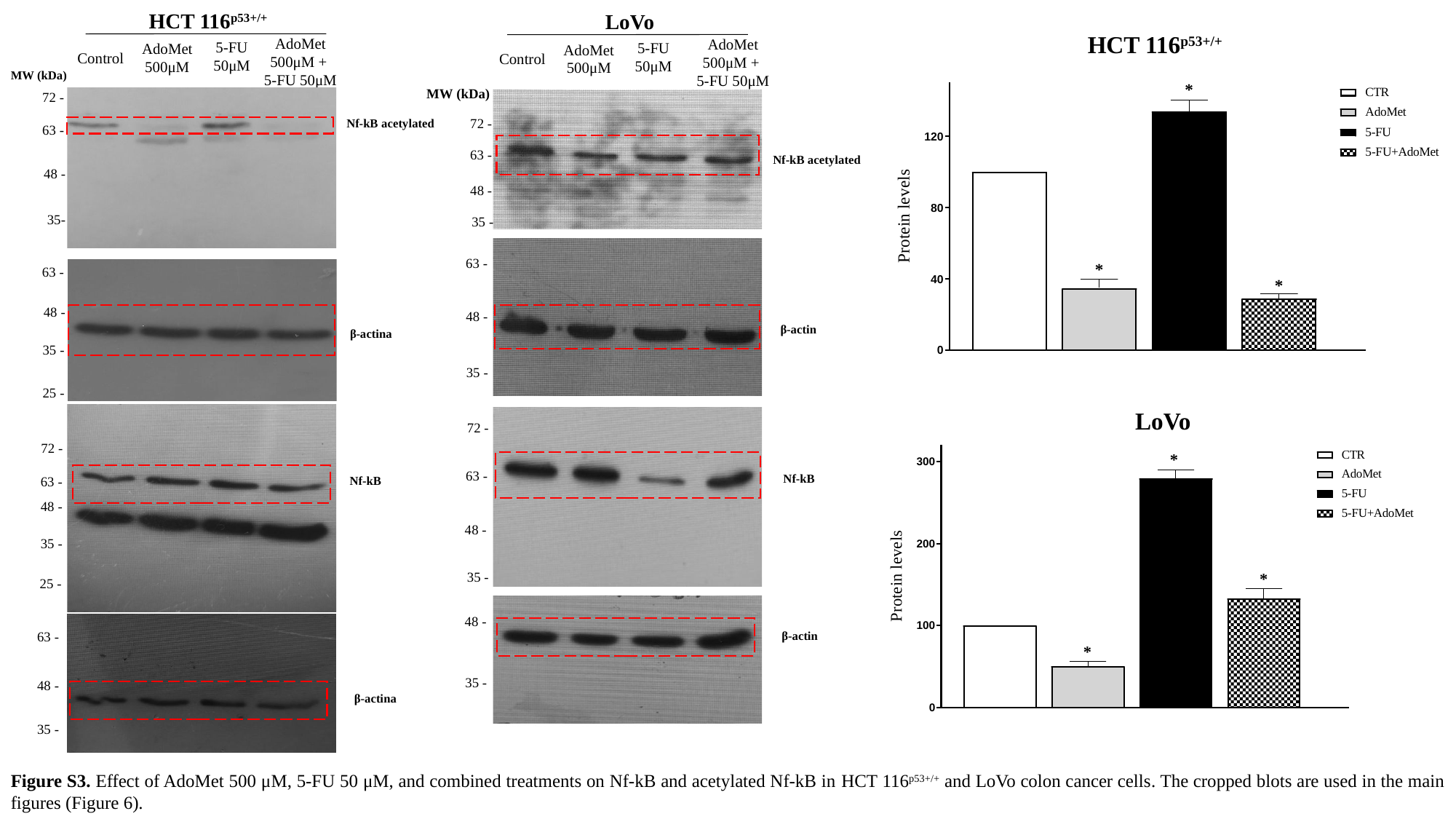

HCT 116p53+/+
LoVo
HCT 116p53+/+
AdoMet 500μM +
5-FU 50μM
AdoMet 500μM +
5-FU 50μM
5-FU 50μM
5-FU 50μM
AdoMet 500μM
AdoMet 500μM
Control
Control
MW (kDa)
MW (kDa)
72 -
72 -
Nf-kB acetylated
63 -
63 -
Nf-kB acetylated
48 -
48 -
35-
35 -
63 -
63 -
48 -
48 -
β-actin
β-actina
35 -
35 -
25 -
LoVo
72 -
72 -
63 -
Nf-kB
63 -
Nf-kB
48 -
48 -
35 -
35 -
25 -
48 -
63 -
β-actin
35 -
48 -
β-actina
35 -
Figure S3. Effect of AdoMet 500 μM, 5-FU 50 μM, and combined treatments on Nf-kB and acetylated Nf-kB in HCT 116p53+/+ and LoVo colon cancer cells. The cropped blots are used in the main figures (Figure 6).
